# Assessment of Molecularly Imprinted Polymers as Selective Solid-Phase Extraction Sorbents for the Detection of Cloxacillin in Drinking and River Water

**DOI:** 10.3390/polym15214314

**Published:** 2023-11-03

**Authors:** Rosa Mª Garcinuño, Eduardo José Collado, Gema Paniagua, Juan Carlos Bravo, Pilar Fernández Hernando

**Affiliations:** Department of Analytical Science, Faculty of Science, Universidad Nacional de Educación a Distancia, Las Rozas, 28232 Madrid, Spain; ejcollado@ccia.uned.es (E.J.C.); gpaniagua@ccia.uned.es (G.P.); juancarlos.bravo@ccia.uned.es (J.C.B.); pfhernando@ccia.uned.es (P.F.H.)

**Keywords:** cloxacillin, molecularly imprinted polymer, solid-phase extraction, adsorption isotherms, water, HPLC-DAD

## Abstract

This paper describes a new methodology for carrying out quantitative extraction of cloxacillin from drinking and river water samples using a molecularly imprinted polymer (MIP) as a selective sorbent for solid-phase extraction (MISPE). Several polymers were synthesized via thermal polymerization using cloxacillin as a template, methacrylic acid (MAA) as a functional monomer, ethyleneglycoldimethacrylate (EGDMA) as a cross-linker and different solvents as porogens. Binding characteristics of the adequate molecularly imprinted and non-imprinted (NIP) polymers were evaluated via batch adsorption assays following the Langmuir and Freundlich isotherms and Scatchard assays. The parameters related to the extraction approach were studied to select the most appropriate polymer for cloxacillin determination. Using the optimized MIP as the SPE sorbent, a simple sample treatment methodology was combined with high-performance liquid chromatography (HPLC) to analyze cloxacillin residues in drinking and river water. Under the optimum experimental conditions, the MISPE methodology was validated using spiked samples. The linearity for cloxacillin was assessed within the limits of 0.05–1.5 µg L^−1^ and the recovery percentage was higher than 98% (RSD < 4%). The limits of detection and limits of quantification were 0.29 and 0.37 µg L^−1^ and 0.8 and 0.98 µg L^−1^ for drinking and river water, respectively. The selectivity of MIP against other ß-lactam antibiotics with similar structures (oxacillin, cefazoline, amoxicillin and penicillin V) was studied, obtaining a good recovery higher than 85% for all except cefazoline. The proposed MISPE-HPLC methodology was successfully applied for the detection of cloxacillin in drinking water from Canal de Isabel II (Madrid) and river water from the Manzanares River (Madrid).

## 1. Introduction

ß-lactam antibiotics (BLAs) constitute a very important group of antibiotics belonging to the penicillin family, which have been broadly used as antimicrobial agents for almost a century. The increase in the consumption of pharmaceutical products in medicine and the veterinary field for the treatment and prevention of diseases, and to promote the growth of livestock, has contributed to a worrying growth in antibiotic-related problems in the aquatic environment [1]. Antibiotics can enter the aquatic system from different sources, including the widespread manufacturing of medical preparations, direct discharge of animal wastewater or seepage from manured farmland [2], and discharge of effluents from the treatment of communal wastewater containing antibiotics in sewage plants, which persist because of the high stability of some compounds or their metabolites. New studies have shown that antibiotics can be found in drinking water from groundwater [3,4]. The presence of antibiotic residues in foodstuffs, including drinking water, has harmful effects on consumers, including allergic reactions, and it affects the intestinal flora and the appearance and spread of antibiotic resistance [5,6,7,8]. So, monitoring residues of pharmaceuticals such as cloxacillin in environmental water samples is very important to avoid its hazard to human health [9].

At present, several screening tests (microbiological and immunological) are published in the literature to detect the presence of the maximum residue limits of ß-lactams in milk and other tissues [10]. However, these assays are highly time-consuming and expensive, have a high percentage of false positives and, in many cases, quantitative determination is not possible. Currently, analytical investigation of cloxacillin in biological fluids, environmental samples and food requires the use of high-performance liquid chromatography (HPLC) combined with ultraviolet (UV) [11], diode array detector (DAD) [12,13], fluorescence detection (FLD) [14], mass spectrometry (MS) [15,16] or tandem mass liquid chromatography coupled to tandem mass spectrometry (LC-MS/MS) [17,18,19,20], which is considered the preferred detection method for routine analysis in a complex matrix because of its great sensitivity and selectivity. Although these methods are rather efficient for the determination of cloxacillin in different matrices, some of them have disadvantages such as low sensitivity, time-consuming sample pretreatment, high-cost instrumentation and difficulties in sample preparation [16,17,19]. The analysis of antibiotics in different matrices usually requires clean-up and preconcentration of the sample. In that sense, solid-phase extraction (SPE) with different types of commercial sorbents and conditions is the most frequently used extraction method for water samples [3,21,22,23,24,25]. The use of molecular imprinting technology for sample preconcentration and efficient clean-up in food and environmental applications is constantly increasing [26,27,28,29]. Most of the present research in the molecular imprinting field is focused on SPE, which offers advantages such as the predetermined selectivity for the analyte(s) of interest, the relatively low amount of solvent and the stability of imprinted polymers, which affect the recovery and contribute highly to the low price of the analysis. These advantages make MISPE technology highly suitable for application to the analysis of antibiotics instead of SPE using commercial cartridges, which, in addition to their high cost, do not allow us to obtain the required selectivity or desired recoveries in some cases, as shown in the study by Bondi et al. [30]. Some MIPs synthesized for cloxacillin were applied to the determination of this antibiotic in milk [31,32], shrimp [33] and other food samples [34]. However, to date, there are no works reported in the literature that address the application of cloxacillin MIPs for water or environmental analyses.

This research describes the assessment of several synthetized MIPs for use as a solid-phase extraction sorbent (MISPE) applied to the quantitative detection of cloxacillin from drinking and river water samples. The developed MISPE-HPLC-DAD methodology was optimized for the detection and quantification of cloxacillin using fortified drinking and river water samples.

## 2. Experiment

### 2.1. Chemical, Reagents and Samples

All reagents were of analytical grade. For the synthesis of MIP, commercial antibiotics standards (cloxacillin, amoxicillin, penicillin V, oxacillin and cefazoline), ethyleneglycoldimethacrylate (EGDMA), 2,2′-azobisisobutyronitrile (AIBN) and methacrylic acid (MAA) were purchased from Sigma-Aldrich (Madrid, Spain). HPLC-grade acetonitrile (ACN), methanol (MeOH) and toluene were supplied by LAB-SCAN (Gliwice, Poland). Deionized water (18.2 MΩ cm) was procured from a Milli-Q water system (Millipore Ibérica, Barcelona, Spain).

Drinking water was collected from Canal de Isabel II in Madrid, which is the Spanish public company that supplies water for human consumption in Madrid. River water was obtained from the Manzanares River (Madrid, Spain).

### 2.2. Chromatographic Conditions

HPLC experiments were performed using a high-performance liquid chromatography instrument 1200 Series from Agilent Technologies (Agilent Technologies, Waldbronn, Germany) equipped with a 1260 diode-array detector, a 1260 quaternary pump and manual injector with a 20 μL sample loop. An analytical column packed with 3 μm C18-Pentafluorophenyl (15 cm × 4.6 mm) from ACE (Advanced Chromatography Technologies, Aberdeen, Scotland) was employed to perform the separation of the analytes. The mobile phase used combines water (solvent A) and acetonitrile (solvent B). The gradient elution was: 5 to 22% B at 0.4–0.7 mL min^−1^ (6 min), 22 to 10% B at 0.7–0.25 mL min^−1^ (0.5 min), 10% B at 0.25 mL min^−1^ (3.5 min), 10 to 22% B at 0.25–0.9 mL min^−1^ (0.5 min), 22% B at 0.9 mL min^−1^ (5.5 min). To achieve maximum sensitivity for cloxacillin determination, the detection wavelength was studied to determine the best quantitative peak areas employing a wavelength of 210 nm. Quantification of the analytes was achieved via external calibration and measurements of the peak area.

### 2.3. Synthesis of Cloxacillin Molecularly Imprinted Polymers

Three cloxacillin-imprinted polymers were synthesized via thermal polymerization. In a glass test tube, we mixed 0.2 mmol of template (cloxacillin), 1.6 mmol of functional monomer (MAA) and 8 mmol of cross-linker (EGDMA). The chemicals were dissolved in a volume of 4.6 mL of different porogens (water, ACN or toluene), with a template:functional monomer:cross-linker molar composition of 1:8:40. After mixture homogenization, 36.3 mg of AIBN (2.0% *w*/*w*) as the free radical initiator was added to the solution. The mixture was purged under a nitrogen stream (5 min) and the glass tube was sealed. Then, the polymerization was carried out at 60 °C in a water bath for 4 h. For reference, non-imprinted polymers (NIPs) were synthesized using the above-described protocol, in which the template molecule was not added to the polymerization mixture. The obtained polymer was ground and wet-sieved with acetone using a metal sieve (355 μm). The template was eliminated from the polymeric matrix using microwave-assisted extraction as the microwave energy provides fast extraction of analytes from solid matrices. The efficiency of this technique is comparable to classical extraction techniques such as Soxhlet or sonication [35]. The procedure used for template removal was that from a previous work [36] (1.5 mL of MeOH: acetic acid (95/5, *v*/*v*) at 800 W, 100 °C, 200 psi with stirring for 20 min). To complete the extraction of the template, this procedure was repeated 3 times, until cloxacillin was not detected in the extraction solvent during HPLC analysis.

### 2.4. Batch Binding Studies

The binding properties of the optimum selected polymer were studied using batch adsorption experiments and Scatchard analysis. The general procedure was as follows: 20 mg of MIP or NIP was incubated for 24 h in the dark in a screw-capped flask with 2.5 mL of water containing cloxacillin at various concentrations (0–300 mg L^−1^). After incubation, the vials were centrifuged at 1200 rpm for 10 min and the free cloxacillin remaining in the supernatant was analyzed using HPLC-DAD at 210 nm.

### 2.5. MISPE Procedure

An amount of 100 mg of polymer was placed in SPE cartridges (3 mL) (Scharlau, Barcelona, Spain) between 2 polyethylene frits with 0.22 μm micropores (Supelco Analytical, Bellefonte, PA, USA). The SPE column containing the cloxacillin MIP was preconditioned with 2 mL of MeOH and next with 1 mL of water before loading 1 mL of sample. The selective washing step was performed with 1 mL of toluene. Finally, the target analyte adsorbed on the polymer was eluted with 1.5 × 3 mL of MeOH. The fractions were collected and evaporated to dryness at room temperature under a nitrogen stream. The residues obtained were reconstituted in a volume of 0.5 mL of water prior to HPLC analysis. The same procedure was used to evaluate the NIP.

## 3. Results and Discussion

### 3.1. Synthesis of Molecularly Imprinted Polymers and Binding Site Evaluation

The MIPs were synthesized following a non-covalent approach using bulk polymerization, as described in Section 2.3. As the functional monomer, methacrylic acid was used due to its capacity to form both ionic and hydrogen bonds with several functional groups from the template. This procedure allowed us to generate specific imprints inside the polymer matrix network. The molar ratio of functional monomer/template is crucial for obtaining specific binding sites. When an excess of monomer is used, elevated non-specific binding sites could be generated in the polymeric matrix [37]. The bifunctional cross-linker monomer EGDMA is mostly used to give a certain mechanical stability in the polymer, instilling appropriate rigidity in the matrix in a molar ratio of 1:5 (monomer:cross-linker). AIBN at 2.0% (*w*/*w*) was used as the heat-induced initiator for the thermal polymerization at 60 °C. The solvent is a key aspect in the polymerization due to the competitive effect with the monomer and the template, independently of the interaction established between these last ones. In this work, three polymers in different media (water, acetonitrile and toluene) were synthetized.

The selection of the most adequate MIP to be used as a selective adsorbent in the SPE process for the determination of cloxacillin was carried out studying the extraction factors, calculated with Equation (1).

(1) $$\mathrm{Extraction}\;\%=\frac{C_{i}-C_{f}}{C_{i}}\;\times \;100$$

C_i_ and C_f_ refer to the concentrations of cloxacillin in the solution before and after extraction, respectively.

To obtain the extraction factor data, the following procedure was performed: 1 mL of cloxacillin solution in water at 1 mgL^−1^ was passed through the SPE cartridge. Previously, a conditioning step was carried out with 2 mL of MeOH and next with 1 mL of water. The fractions of cloxacillin collected were analyzed using HPLC-DAD. Data obtained showed that the extraction factor for the water MIP was 54%, for the toluene MIP was 75% and for the ACN-MIP was 94%; meanwhile, the NIP extraction factors in these solvents were 16, 14 and 21%, respectively, lower that those obtained for the MIPs. This study showed that ACN was the optimum porogen for the MIP synthesis due to the strong affinity of ACN-MIP for cloxacillin and the good specificity obtained compared with the non-imprinted polymer (ACN-NIP). According to obtained results, ACN-MIP was chosen as the optimum sorbent in the SPE procedure to carry out cloxacillin determination.

### 3.2. Saturation Binding Curves and Scatchard Plot Analysis

**Figure 1:**
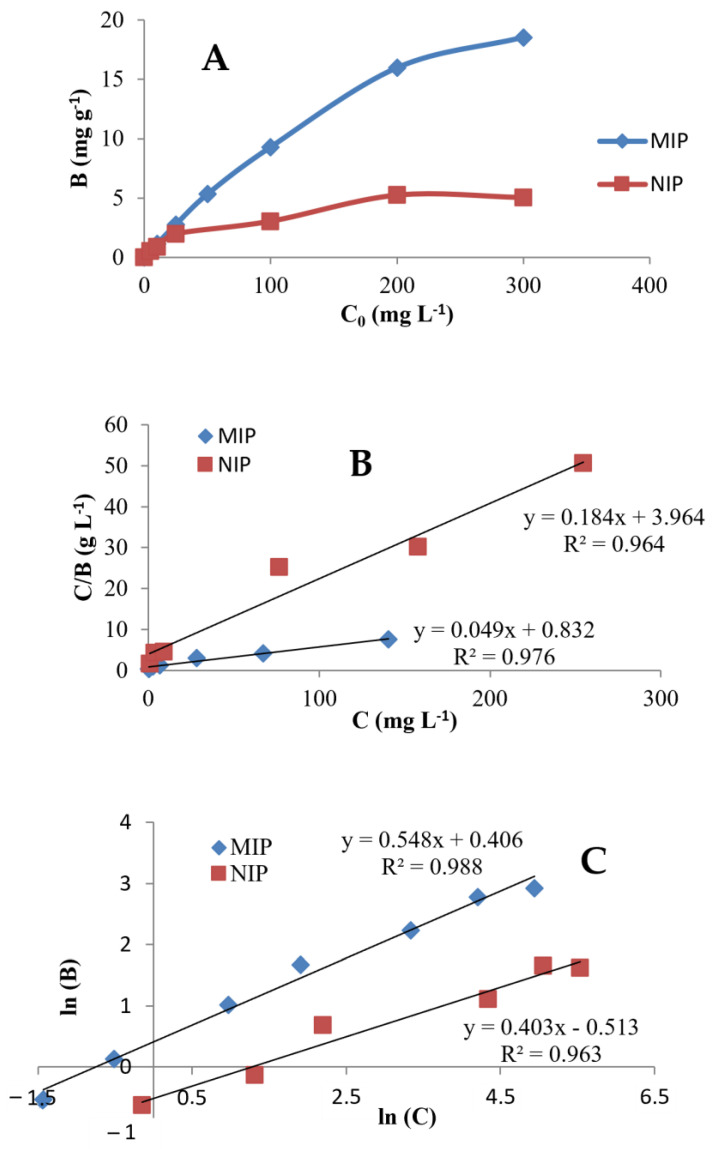
(**A**) Adsorption isotherm of MIP and NIP using batch adsorption assay. (B) is the binding of cloxacillin per g of polymer, (*C*_0_) is the initial cloxacillin in the medium. (**B**) Langmuir isotherms linearized for cloxacillin adsorption onto MIP and NIP. (**C**) Freundlich isotherms linearized for cloxacillin adsorption onto MIP and NIP.

The binding affinity of ACN-MIP for cloxacillin was evaluated using binding isotherm experiments and Scatchard plot analysis. Batch adsorption experiments were carried out as follows: 2.5 mL of solutions of cloxacillin at a concentration ranging from 0 to 300 mg L^−1^ were incubated with 20 mg of ACN-MIP or ACN-NIP for a period of 24 h. Then, the concentration of the remaining cloxacillin in the solution was quantified via chromatographic detection at 210 nm. Figure 1A represents the binding of cloxacillin per g of polymer (B), for both imprinted and non-imprinted polymers, versus the initial concentration of cloxacillin in the medium ($C_{0}$). The cloxacillin equilibrium concentration bound to ACN-MIP increases with the initial cloxacillin concentration in solution. The amount of cloxacillin bound to ACN-MIP is higher than the amount of cloxacillin bound to ACN-NIP, which means higher affinity of ACN-MIP to cloxacillin due to the specific sites generated in the synthesis.

Different adsorption isotherm models have been applied to characterize the sorption behavior of MIPs. Langmuir and Freundlich isotherms are the most commonly used models and can be utilized to determine sorbent properties such as adsorption mechanism, surface area and affinities with the target, as these isotherms show the distribution of molecules between the liquid and solid phases at the equilibrium time. Experimental results fitted to Langmuir and Freundlich isotherms are included in Table 1.

The main premise assumed in the Langmuir isotherm model is that the sorbent is monolayer, where the sites only take one molecule of the target each, making the adsorption energy constant over all sites. When every adsorption site has sorbed one target molecule, the maximum surface adsorption is achieved and no more sorption can take place [38]. The obtained data on ACN-MIP and ACN-NIP were fitted to the Langmuir adsorption model using Equation (2):

(2) $$\frac{C}{B}=\left(\frac{1}{B_{0}\times K_{L}}\right)+\left(\frac{C}{B_{o}}\right)$$

In Equation (2), C (mg L^−1^) refers to the remaining analyte concentration at equilibrium, B (mg g^−1^) refers to the cloxacillin amount bound to ACN-MIP or ACN-NIP (g) at equilibrium, $B_{0}$ (mg g^−1^) refers to the maximum amount of cloxacillin related to complete coverage of the monolayer on the surface and $K_{L}$ (L mg ^−1^) refers to the Langmuir constant, which is associated with adsorption energy. The experimental data were plotted in Figure 1B, showing a linear relationship where the Langmuir constants $B_{0}$ and $K_{L}$ can be calculated with Equation (2). The maximum monolayer capacities, $B_{0}$, were 0.25 mg g^−1^ and 1.21 mg g^−1^ for ACN-MIP and ACN-NIP, respectively, while the Langmuir constants $K_{L}$ were 80.2 mg L^−1^ and 4.51 mg L^−1^ for MIP and NIP, respectively (Table 1).

The Langmuir model is restricted to a monolayer, while the Freundlich isotherm model corresponds to a heterogeneous adsorbent surface caused by multilayer adsorption and an exponential distribution of the active sites and their energies. The empirical equation that represents the Freundlich adsorption isotherm is as follows (3):

(3) $$\ln\left(B\right)=\ln\left(K_{F}\right)+\frac{1}{n}ln(C)$$

In Equation (3), $K_{F}$ (mg g^−1^) refers to a Freundlich constant corresponding to the binding energy and n (dimensionless) is a Freundlich constant representing the heterogeneity index according to this empirical model. The adsorption is linear when n=1, a chemical process when n<1 and a physical process when n>1 [39]. Figure 1C shows the graph of $\ln\left(B\right)$ versus ln⁡(C) using binding data from ACN-MIP and ACN-NIP, which give a straight line. The values of Freundlich constants $K_{F}$ were 1.5 mg g^−1^ for MIP and 0.5 mg g^−1^ for ACN-NIP, while the n constants were 1.8 and 2.5 for ACN-MIP and ACN-NIP, respectively. The value of n for ACN-MIP was lower than for ACN-NIP, which means that ACN-MIP has a higher percentage of binding sites of high affinity.

To finish the evaluation of the binding characteristics between cloxacillin and polymer at equilibrium and predict the affinity between the solid phase and the analyte, the $R_{L}$ parameter (dimensionless) was studied. $R_{L}$, which describes the nature of the adsorption process, is represented in Equation (4), where $K_{L}$ (L mg ^−1^) refers to Langmuir constant and $C_{0}$ (mg L^−1^) refers to the beginning analyte concentration.

(4) $$R_{L}=\frac{1}{1+(K_{L}\times C_{0})}\;\;$$

The type of Langmuir isotherm is defined by the value of $R_{L}$: irreversible when $R_{L}=0$, favorable when $R_{L}<1$, linear when $R_{L}=1$ or unfavorable when $R_{L}>1$ [40]. The $R_{L}$ obtained for ACN-MIP and ACN-NIP were all positive and less than 1, between 0.001 and 0.04, indicating that the adsorption process of cloxacillin was favorable.

The Scatchard plot is used to determine the number and type of binding sites of the polymers. This estimated model is often used in solid-phase extraction to characterize the maximum amount of adsorption and the dissociation constant. The binding data were linearly transformed according to the Scatchard equation [39].

(5) $$\frac{B}{C}=\frac{\left(B_{\max}-B\right)}{K_{d}}$$

**Figure 2:**
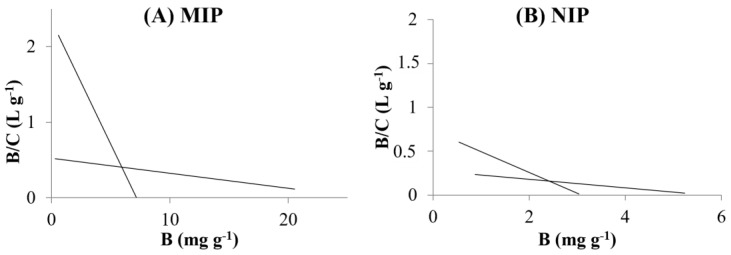
Scatchard plots of the binding of cloxacillin to the MIP (**A**) and to the NIP (**B**).

In Equation (5), B (mg g^−1^) refers to cloxacillin bound to 1 g of ACN-MIP or ACN-NIP at equilibrium, C (mg L^−1^) refers to the remaining analyte concentration at equilibrium, $B_{\max}$ (mg g^−1^) refers to the maximum amount of adsorption of cloxacillin, and $K_{d}$ (mg L^−1^) refers to the equilibrium dissociation constant of the binding sites. The slope and the intercept of the plot B/C versus B correspond to $K_{d}$ and $B_{\max}$, respectively. The Scatchard plots of ACN-MIP and ACN-NIP (Figure 2A and 2B, respectively) are both nonlinear.

The data could be fitted to two straight lines with different slopes, which means the existence of heterogeneous binding sites, both in ACN-MIP and ACN-NIP, representing binding sites of high and low affinity. The dissociation constant of the binding sites ($K_{d}$) and the apparent maximum number of the binding sites $(B_{\max}$) could be obtained from the slope and the intercept, respectively. Table 2 shows the outcomes of Scatchard assays for both polymers ACN-MIP and ACN-NIP. In ACN-MIP, the values of $B_{\max}$ and $K_{d}$ are 2.9 mg g^−1^ and 1.3 mg L^−1^, respectively, for binding sites of high affinity and 22.6 mg g^−1^ and 57.2 mg L^−1^, respectively, for the low-affinity binding sites. Meanwhile, the ACN-NIP results revealed values of 1.8 mg g^−1^ and 2.7 mg L^−1^ for $B_{\max}$ and $K_{d}$ (high-affinity binding sites) and 6.1 mg g^−1^ and 181.6 mg L^−1^ (binding sites of low affinity), respectively. These results show that ACN-MIP had bigger $B_{\max}$ and lower $K_{d}$ in ACN-MIP than ACN-NIP, due to the specific binding sites.

The synthesis of polymers via the non-covalent method could explain the heterogeneity of the surface of the MIP. Although this method generates specific binding sites, some interactions between the template and the functional monomers cannot be made properly. This incompleteness could generate non-specific binding sites, causing the heterogeneous surface.

### 3.3. MISPE Procedure Optimization

**Figure 3:**
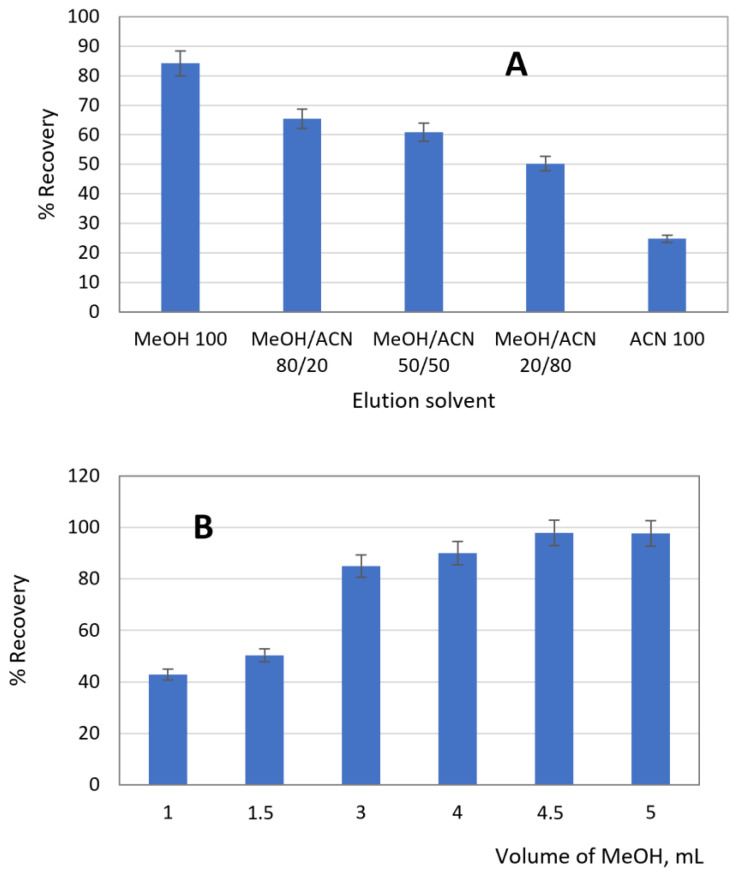
Optimization of MISPE. (**A**) Solvent elution. (**B**) Volume of MeOH as optimum solvent elution.

The MISPE procedure was optimized using ACN-MIP as the sorbent to obtain the best selectivity and recovery conditions for cloxacillin. Firstly, the conditioning stage was evaluated using MeOH (3 × 1 mL) or 2 mL of MeOH and 1 mL of Milli-Q water and, subsequently, a solution of cloxacillin in drinking water at 1 mg L^−1^ was loaded onto the SPE column. The higher retention of cloxacillin (96%) occurred after conditioning the column with 2 mL MeOH and 1 mL of Milli-Q water, being 76% when only MeOH was used. To optimize the washing step, different solvents (water, acetonitrile and toluene) were tested. Water and acetonitrile washed cloxacillin from the polymer. Given the low solubility of cloxacillin in toluene, this solvent was chosen to wash the non-specifically nonpolar bindings. To achieve a quantitative extraction of cloxacillin from the MISPE column, different volumes (1–5 mL) of MeOH and ACN or MeOH/ACN mixtures were examined as eluents. Recoveries were calculated from the fractions collected after the elution step, taking into account the amount of amoxicillin retained on the column in the loading step. Figure 3A shows the elution capacity of the solvents tested when 3 mL (2 × 1.5) was used. It is shown that recoveries increased with the increment of methanol percentage, reaching the maximum when 100% methanol was used. The effect of solvent volume on the elution process was tested (Figure 3B). The complete elution of cloxacillin was reached with MeOH (3 × 1.5 mL).

### 3.4. Validation and Applicability of the MISPE-HPLC Methodology

The MISPE method developed for cloxacillin was validated by testing analytical parameters such as linearity, accuracy, precision and limits of detection and quantification using spiked water samples. River water required a previous filtration step through a PTFE filter (0.2 μm) to remove suspended matter before being submitted to an optimized MIPSE procedure.

Linearity was evaluated using spiked water samples with increasing concentrations of cloxacillin in the range of 0.05–1.5 µg L^−1^. Calibration curves were plotted by means of the cloxacillin concentration versus peak area. The obtained results showed good linearity in both drinking and river water (R^2^ > 0.998). The accuracy and precision of the developed method were assessed via analysis of drinking and river water samples spiked at three concentration levels (0.1, 0.5, 1 µg L^−1^). Samples were analyzed in quintuplicate for the intra-day study and in triplicate for the inter-day and accuracy studies. Standard deviations were calculated in all cases (Table 3). The % RSD values showed good precision of the developed method. The obtained values for the detection limit (LOD), calculated as a signal-to-noise ratio of 3, were 0.29 and 0.37 µg L^−1^ for drinking and river water, respectively. The quantification limits (LOQs), calculated as a signal-to-noise ratio of 10, were 0.8 and 0.98 µg L^−1^ for drinking and river water, respectively.

The comparison of the developed method with other reported techniques for the determination of cloxacillin, such as the detection method, extraction method, LOD, LOQ and recovery, is summarized in Table 4.

### 3.5. Selectivity of the MIP

**Figure 4:**
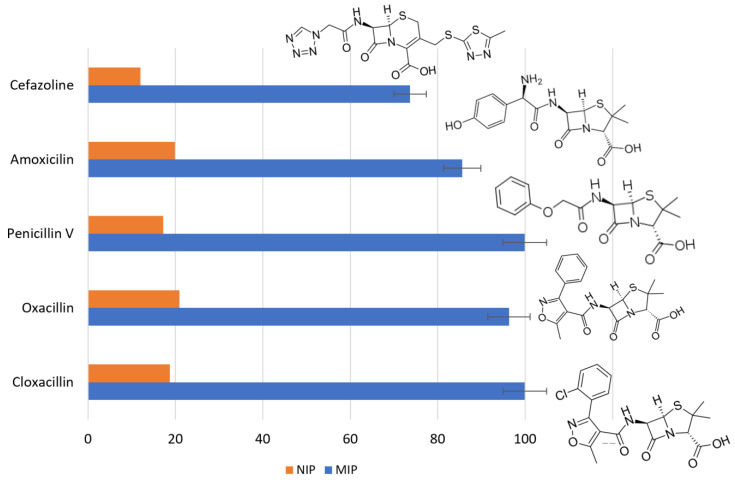
Recoveries of the ß-lactam antibiotics at 1 µg L^−1^.

Once the MISPE procedure was optimized, the selective adsorption of the ACN-MIP towards other ß-lactam antibiotics structurally analogous to cloxacillin such as amoxicillin, penicillin V, oxacillin and cefazoline was evaluated. The recovery percentages were calculated considering the fractions collected after the elution step with respect to the initial concentration of each compound loaded onto the SPE column. The results obtained are summarized in Figure 4. Recoveries for cloxacillin, penicillin V, oxacillin and amoxicillin were higher than 85%, while the recovery for cefazoline was approximately 74%. The results were in accordance with the chemical structures of the compounds assayed. This study revealed a high adsorption capacity of ACN-MIP towards the ß-lactam antibiotics with similar chemical structures to cloxacillin (molecule template) in comparison with cefazoline, the compound that most differs structurally from cloxacillin, which means that residues of five ß-lactam antibiotics could be extracted using the developed MISPE methodology. Furthermore, the adsorption capacity of the antibiotics onto ACN-NIP was evaluated. The obtained results showed a retention capacity lower than 22% for all the analytes.

## 4. Conclusions

The use of a molecularly imprinting polymer was evaluated to develop an SPE method for the detection of cloxacillin in drinking and river water samples via HPLC-DAD. Binding properties of the polymer selected as optimum were studied using the Langmuir and Freundlich models and Scatchard analysis. Imprinted and non-imprinted polymers synthesized in ACN, ACN-MIP and ACN-NIP are well-described by these models with high- and low-affinity binding sites. Synthetized MIP exhibited a very high adsorption capacity for cloxacillin and good selectivity. The specific recognition of MIP towards other antibiotics belonging to the family of ß-lactams was tested. The obtained results showed successful recovery of antibiotics with a similar chemical structure (amoxicillin, penicillin V and oxacillin) and lower recovery for cefazoline because of lesser structural similarity. Hence, our work revealed that the obtained ACN-MIP has significant potential for use as specific sorbent in the SPE method for the analysis of five beta-lactams in drinking and river waters at trace levels. The developed MISPE-HPLC methodology offers a rapid, easy and reliable alternative procedure, consuming low volumes of solvents. This work thus makes a significant advance in water quality control with respect to other published methods due to the simplicity, low cost and sensitivity of our method.

## Figures and Tables

**Table 1:** Langmuir and Freundlich isotherm data for adsorption of cloxacillin on ACN-MIP and ACN-NIP.

Isotherm Results	Langmuir				Freundlich		
	$B_{0}$ (mg g^−1^)	$K_{L}$ (L mg^−1^)	$R_{L}$	$R^{2}$	KF (mg g^−1^)	n	$R^{2}$
ACN-MIP	0.25	80.2	0.001	0.964	1.5	1.8	0.988
ACN-NIP	1.21	4.51	0.004	0.976	0.5	2.5	0.963

**Table 2:** Results of Scatchard analysis for ACN-MIP and ACN-NIP.

Binding Sites	ACN-MIP		ACN-NIP	
	$B_{\max}$ (mg g^−1^)	$K_{d}$ (mg L^−1^)	$B_{\max}$ (mg g^−1^)	$K_{d}$ (mg L^−1^)
High affinity	2.9	1.3	1.8	2.7
Low affinity	22.6	57.2	6.1	181.6

**Table 3:** Analytical characteristics for cloxacillin of the developed MISPE method for drinking and river water.

Sample	Linearity		Spiking Level µg/L	Recovery ± RSD %		LOD µg/L	LOQ µg/L
	Concentration Range µg/L	*R* ^2^		Inter-Day	Intra-Day		
Drinking water	0.05–1.50	0.999	0.10	96.9 ± 6.3	90.5 ± 7.5	0.29	0.8
			0.50	94.7 ± 4.2	92.5 ± 6.8		
			1.00	93.8 ± 6.4	91.8 ± 7.9		
River water	0.05–1.50	0.999	0.10	83.1 ± 2.2	89.9 ± 6.0	0.37	0.98
			0.50	84.3 ± 5.2	80.5 ± 5.0		
			1.00	89.1 ± 4.2	81.7 ± 7.7		

**Table 4:** Comparison of the proposed method with other published methods for determination of cloxacillin.

Sample Matrix	Extraction Method	Detection Method	Recovery (%)	LOD	LOQ	Ref.
Milk	SPE	Electrochemical sensor	98.6–101.8	36 nM		[31]
Shrimp	MIM	HPLC-UV	80.9–94.9	0.03 μg/g	0.10 μg/g	[33]
Pig plasma	MMIP	SERS	<80.0	7.80 pmol		[34]
Drinking water Tap water	MISPE	HPLC-DAD	93.8–96.9	0.29 µg/L	0.80 µg/L	Present work
			83.1–89.1	0.37 µg/L	0.98 µg/L	

MIM: molecularly imprinted membranes; MMIP: magnetic molecularly imprinted polymer; SERS: surface-enhanced Raman spectroscopy.

## Data Availability

Data are contained within this article.
